# Glymphatic Drainage Blocking Aggravates Brain Edema, Neuroinflammation *via* Modulating TNF-α, IL-10, and AQP4 After Intracerebral Hemorrhage in Rats

**DOI:** 10.3389/fncel.2021.784154

**Published:** 2021-12-17

**Authors:** Xichang Liu, Gang Wu, Na Tang, Li Li, Cuimin Liu, Feng Wang, Shaofa Ke

**Affiliations:** ^1^Department of Neurology, First People’s Hospital of Yichang, Yichang, China; ^2^Department of Pharmacy, Taizhou Hospital of Zhejiang Province Affiliated to Wenzhou Medical University, Linhai, China; ^3^Department of Neurology, Taizhou Hospital of Zhejiang Province Affiliated to Wenzhou Medical University, Linhai, China

**Keywords:** glymphatic, intracerebral hemorrhage, aquaporins-4, neuronal apoptosis, neuroinflammation, brain edema

## Abstract

**Objective:** The “Glymphatic” system, a network of perivascular tunnels wrapped by astrocyte endfeet, was reported to be closely associated with the diseases of the central nervous system. Here, we investigated the role of the glymphatic system in intracerebral hemorrhage (ICH) and its protective mechanism.

**Method:** Experimental ICH model was induced by type IV collagenase in rats. Cerebral lymphatic blockage was induced by ligation and removal of cervical lymph nodes. The experimental rats were divided into sham-operated (SO) group, ICH group, and cerebral lymphatic blocking and ICH (ICH + CLB) group. Neurological scores were measured using the Garcia scoring system on the third and seventh day after ICH. Active caspase-3 was immunostained to evaluate neuronal apoptosis. Brain water content was calculated using the dry-wet specific gravity method. The expression of inflammatory factors TNF-α, IL-1β, and IL-10 were detected using ELISA. Aquaporins-4 (AQP-4) and glial fibrillary acidic protein (GFAP) were detected using western blot analysis.

**Results:** The neurological scores of rats in the CLB + ICH group were significantly lower than those in the in ICH group. The number of active caspase-3 neurons was significantly higher in the CLB + ICH group compared to the ICH group. CLB significantly aggravated ICH-induced brain edema 3 d after ICH. There was an increase in the expression of TNF-α, IL-1β, IL-10, AQP-4, GFAP after ICH. The expression of TNF-α was significantly higher in the CLB + ICH group compared to ICH group 3 d after ICH while there was no difference 7 d after ICH. There was no statistical difference in the expression of IL-1β between the ICH group and CLB + ICH group. However, the expression of IL-10 in the CLB + ICH group was significantly lower than that in the ICH group. Lastly, AQP-4 expression was significantly lower in the CLB + ICH group compared to the ICH group while the expression of GFAP was higher in the CLB + ICH group compared to the ICH group.

**Conclusion:** CLB exacerbated cerebral edema, neuroinflammation, neuronal apoptosis and caused neurological deficits in rats with ICH *via* down-regulating AQP-4, up-regulating inflammatory TNF-α and inhibiting IL-10 expression. The glymphatic drainage system protects against neurologic injury after ICH induction in rats under normal physiological conditions.

## Introduction

Nearly two million patients develop intracerebral hemorrhage (ICH) in a year, with more than half of the ICH survivors suffering from neurological dysfunction (Del Brutto, 2017). In the recent two decades, the incidences of long-term disability and mortality associated with ICH have remained unchanged (Kitagawa, 2014). This suggests that more work is needed to improve ICH treatment and reduce the risk of death. ICH is associated with toxic metabolites in the hematoncus, which leads to neuronal dysfunction and neurologic impairment (Song et al., 2020). As for the absorption and excretion pathways of these toxic metabolites, the traditional view is that hemosiderin and necrotic brain tissues are mostly engulfed by phagocytes (Chen C. et al., 2020). However, some soluble macromolecular wastes such as amyloid are not able to cross the blood-brain barrier and enter circulation (Carare et al., 2008).

Recent studies on drainage pathways using tracer techniques in different species including humans have uncovered roles for an avascular “glymphatic” system in the central nervous system (CNS) (Visanji et al., 2018; Goodman and Iliff, 2019). Macromolecular substances in the brain are filtered into the CSF through gaps around arterial blood vessels in the outer membrane and extracellular space. These substances are exchanged with ISF through Aquaporins-4 (AQP-4) and ion concentration gradient, and then flow into cervical lymph nodes through peri-venous spaces and dura lymphatic capillaries, and finally drain back into the cervical vein (Wu et al., 2020). As a result, the system has developed unique adaptations for waste clearance and fluid balance, and is known as the “glymphatic” system (Plog and Nedergaard, 2018).

The glymphatic system is a network of perivascular tunnels wrapped by astrocyte endfeet (Hablitz et al., 2020) and aquaporin-4(AQP-4), a water channel protein mainly in the perivascular astrocyte endfeet, plays an important role in the exchange of cerebrospinal fluid (CSF) with interstitial fluid (ISF) (Nakada and Kwee, 2019). Recent studies have identified the “glymphatic” system as a novel therapeutic target that is closely associated with central nervous system degeneration diseases, such as multiple sclerosis, ischemic stroke, and Alzheimer’s disease (Lin et al., 2020; Reeves et al., 2020). In this study, we investigated the effect of the glymphatic pathway on neurological function and its protective mechanism in rats with ICH. The parameters investigated include cerebral edema, neuroinflammation, and neuronal apoptosis, and AQP-4.

## Materials and Methods

### Animals

A total of 75 male Sprague–Dawley rats (age 8–10 weeks; weight 280–320 g; Shanghai Slac Laboratory Animal Co., Ltd.) were used in the study. The rats were housed in temperature and humidity-controlled animal quarters with a 12-h light/dark cycle. All experimental protocols and animal handling procedures were reviewed and approved by the Committee for Animal Experiments at Taizhou Hospital of Zhejiang Province in China. The rats were divided into sham-operated (SO) group (*n* = 25), ICH group (*n* = 25), and cerebral lymphatic blocking (CLB) and intracerebral hemorrhage (ICH + CLB) group (*n* = 25).

### Rat Cerebral Lymphatic Blocking Model

The rats were anesthetized using sodium pentobarbital (55 mg/kg) and then immobilized on the dissection surface in the supine position. The neck skin was cleaned, sterilized with iodophor, and then cut approximately 2 cm lengthwise along the midline of the neck. Cervical subcutaneous tissue was separated using a blunt dissector. The submaxillary lymph nodes (about 2–3 on each side) located in the anterolateral side of the submandibular gland, superficial cervical lymph nodes (about 1–2 on each side) located in the bifurcated external jugular vein and deep cervical lymph nodes (1 or 2 on each side) located in the middle of the sternocleidomastoid were exposed and removed. The lymphatic input and output vessels were then ligated with sterile surgical sutures. Lastly, the incisions on the subcutaneous tissue and skin were sutured layer by layer.

### Rat Intracerebral Hemorrhage Model

The experimental ICH model was generated as described in our previous study (Liu et al., 2016). Briefly, rats were anesthetized using sodium pentobarbital (55 mg/kg) and then fixed in the stereotactic frame facing downward. During the surgical procedure, the rectal temperature of the rats was maintained at 37 ± 0.5°C using a rectal thermostat probe and heating pad. In addition, the heart rate and arterial blood pressure of the rats were monitored using a Data Acquisition Systems (AD Instruments, Milford, MA, United States). A burr hole with a diameter of 3 mm was carefully drilled using an electric drill along the right coronal suture at 3.0 mm lateral and 0.5 mm posterior to the bregma. A 30-gauge (G) needle was inserted into the right caudatum with its tip 6 mm beneath the dural surface. Afterward, 0.3U collagenase IV (Sigma-Aldrich V900893, United States) in 1.0 μl saline was slowly injected into the right caudatum for 10 min and the needle removed from the caudatum 20 min after injection. The burr hole was sealed with bone wax and the incision on the skin was sutured. For rats in the CLB + ICH group, the ICH model was generated 24 h after generation of the CLB model.

### Measurement of the Neurological Score

The Garcia scoring system was used to determine neurological scores at day (d) 3 and 7 after induction of ICH. Neurological scores were determined by an investigator blinded to the experimental treatment scheme. Several indexes were evaluated including spontaneous activity, symmetrical movements of limbs, forepaw outstretching, climbing, body proprioception, and response to vibrissae touch (Liu et al., 2020). Scoring of each subtest was 0 (worst) to 3 (best). The neurological score of the rats was calculated by summing up the subtest scores.

### Tissue Immunofluorescent Staining

At 3 days after ICH induction, rats were deeply anesthetized through intraperitoneal injection and perfused transcardially in turn with 0.9% saline and 4% paraformaldehyde. The forebrains were dissected out and post-fixed in 4% paraformaldehyde overnight at 4°C, followed by sinking in 20 and 30% sucrose. Tissues were embedded in OTC gel. Brain sections were obtained from the regions 3 mm anterior and 4 mm posterior to the bregma using a cryostat at −18°C. Coronal sections at 10 μm were serially cut using a cryostat (Leica CM1950, Nussloch, Germany). Brain sections were mounted on superfrost plus slides and stained with caspase-3 antibody (Abcam). The sections were incubated with Alexa Fluor488 second antibody (Life Technologies, Carlsbad, CA, United States) and counterstained with DAPI. Five brain sections were selected per mouse and analyzed with a laser-scanning confocal microscope (Zeiss LSM800, München, Germany). Three rats were selected per group for the analysis and the mean number of positive cells per section was recorded.

### Brain Water Content Measurement

At 3 and 7 d after ICH induction, rats from each group (*n* = 5) were anesthetized, and the brain on the side of hematoma quickly removed and weighed, to give the wet weight. The brains were then reweighed after heating in an oven (70°C) for 72 h, to give the dry weight. The percentage of water content was calculated as [(wet weight−dry weight)/wet weight × 100%].

### Assessment of Hematoma Volume

To determine the hematoma volume, the rats were sacrificed after being anesthetized deeply using sodium pentobarbital 7 d after ICH induction. The brain of the rat was perfused, fixed, and serially sliced (2 mm thickness) in the regions anterior and posterior to the needle entry site (Liu et al., 2016). Hematoma volumes were determined by analyzing the serial brain slices using a digital scanner and an image analysis program (Image J, NIH, United States).

### Enzyme-Linked Immunosorbent Assay

At 3 and 7 d after ICH induction, the rats were decapitated, and the brains immediately dissected out (*n* = 5/group). Segments of cerebral cortex surrounding the hematoma (approximately 100 mg) were quickly dissected and stored at −80°C. The tissue was weighted, cut into small pieces, dispersed by aspiration into a pipette, suspended in physiological salt solution in a test tube, and homogenized using a power-driven homogenization device to obtain 10% homogenate. Samples were kept on wet ice for 20 min before further analysis. The homogenates were centrifuged at 11,000 rpm for 10 min and the supernatant was used for detection of TNF-α, IL-1β, IL-10, and MBP (Sinobest Rat Elisa Kit).

### HE Staining

The rats were deeply anesthetized through intraperitoneal injection, followed by the opening of the thoracic cavity using a pair of sterile scissors. The abdominal aorta and inferior vena cava were closed with a vascular clamp. The brains of rats were perfused transcardially in turn with 0.9% saline and 4% paraformaldehyde. The forebrains were dissected out and post-fixed overnight in 4% paraformaldehyde at 4°C. The brains were then dehydrated, paraffin-embedded and cut into 6 μm slices using a microtome (Leica RM2135, Nussloch, Germany). The brains sections were deparaffinized, rehydrated, and stained with hematoxylin for 5 min. Then, the sections were washed under running water for 10 min, and counterstained with eosin for 5 min. After differentiation, the sections were dehydrated sequentially in 70, 95, and 100% ethyl alcohol. The slides were then cleared using xylene and permanently mounted on microscope slides.

### Western Blot Analysis

The perihematomal tissues were extracted and then homogenized in lysis buffer containing a protease inhibitor cocktail. The supernatant was collected after centrifugation at 12,000 rpm for 10 min. The protein concentration was determined using the rapid gold BCA protein assay kit (Thermoscientific, Rockford, United States). Equal amount of protein were separated through 10% SDS PAGE then transferred onto a PVDF membrane (Millipore, Billerica, MA, United States). The blots were incubated overnight at 4°C with anti-AQP4 (ab46182, abcam) and anti-GFAP (MAB3402, Millipore) antibodies. Protein bands were detected using an enhanced chemiluminescence detection system (Cell Signaling Technology, Beverly, MA, United States) and the intensities of immunoreactive bands were analyzed using an image analysis program (Image J, NIH, United States).

### Statistical Analysis

Statistical analysis was carried out using the SPSS statistical package (SPSS 14.0, SPSS, Inc., Chicago, IL, United States). Student’s *t*-test was used when comparing two groups. One-way ANOVA was employed when comparing multiple groups. *Post hoc* analysis was conducted using the Tukey’s *post hoc* test. *P* < 0.05 was considered to be statistically significant.

## Results

### Cerebral Lymphatic Blocking Exacerbated Neurological Impairment After Intracerebral Hemorrhage

**FIGURE 1:**
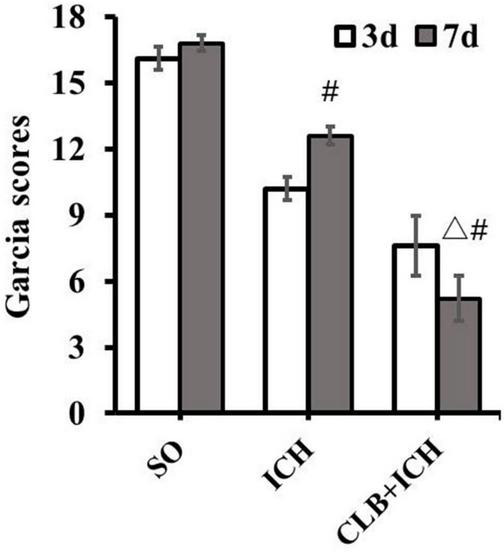
Neurological deficit score after cerebral lymphatic blocking (CLB) in intracerebral hemorrhage (ICH) rats. The scores of the rats in the CLB + ICH group were significantly lower than those of rats in the ICH group 7 days after ICH (△, *P* < 0.05). There was a significant increase in the scores for the rats 7 days after ICH compared to 3 day after ICH (#, *P* < 0.05). On the contrary, there was a decrease in the scores 7 days after ICH compared to 3 days after ICH for rats in the CLB + ICH group (#, *P* < 0.05). SO group, *n* = 10. ICH group, *n* = 10; and CLB + ICH group, *n* = 10, 3, or 7 days after ICH.

Firstly, there was no difference in physiological parameters such as heart rate, mean arterial pressure, temperature and glucose of each group during operation (data not shown). To determine the effect of ICH on neurological impairment, the Garcia scoring system was used for neurological scoring 3 and 7 d after ICH induction. The results showed that there were significant differences in the scores between the SO group (16.5 ± 0.9) and the ICH group (10.2 ± 2.1) at 3 d after ICH (*P* < 0.05, Figure 1). On the other hand, there was a significant decrease in the scores for the rats in the CLB + ICH group compared to the rats in the ICH group at 7 d after ICH (5.6 ± 3.2 vs. 13.6 ± 1.2), *P* < 0.05, Figure 1). There was a significant increase in the neurological scores 7 days after ICH compared to 3 day after ICH (13.6 ± 1.2 vs. 10.2 ± 2.1, *P* < 0.05). However, there was a decrease in the scores in the CLB + ICH group (5.6 ± 3.2 vs. 7.2 ± 2.3, *P* < 0.05, Figure 1). These data indicated that CLB aggravated neurological impairment caused by ICH.

### Cerebral Lymphatic Blocking Exacerbated Neuronal Apoptosis in Peri-Hematoma After Intracerebral Hemorrhage

**FIGURE 2:**
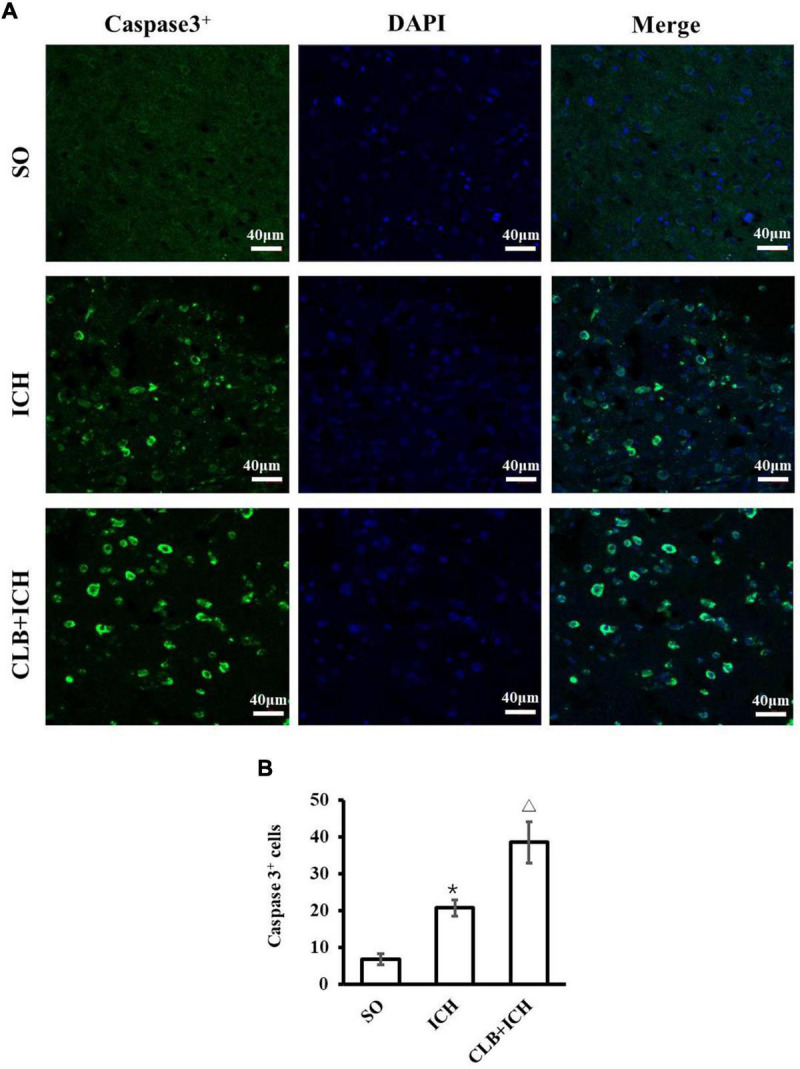
Results of neuronal apoptosis 3 days after ICH in three groups. **(A)** Representative immunofluorescent images of Active caspase-3 (green) and DAPI (blue) staining (×200). Scale bar: 40 μm. **(B)** Quantitative analysis showed more apoptotic cells 3 days in ICH and CLB + ICH groups than in SO group (*P < 0.05). The numbers of active caspase-3cells in hemorrhagic rat brain tissues of CLB + ICH group were further increased comparing with those in ICH group (△, *P* < 0.05). SO group, *n* = 5; ICH group, *n* = 5; and CLB + ICH group, *n* = 5.

In consideration of neurological impairment, active caspase-3 immunostaining was chosen to analyze the level of neuronal apoptosis in peri-hematoma 3 days after ICH (Figure 2A). The number of active caspase-3 neurons were significantly higher in the ICH group compared to that of the SO group (20.7 ± 6.9 vs. 5.2 ± 1.6, *P* < 0.05, Figure 2B). Furthermore, CLB significantly increased the number of active caspase-3 neurons in the CLB + ICH group compared to the ICH group (38.5 ± 7.1 vs. 20.7 ± 6.9, *P* < 0.05, Figure 2B). These data demonstrated that the CLB increases ICH-induced apoptosis of neurons in rats.

### No Change of Hematoma Volume but Deteriorated Brain Edema in Cerebral Lymphatic Blocking + Intracerebral Hemorrhage Group

**FIGURE 3:**
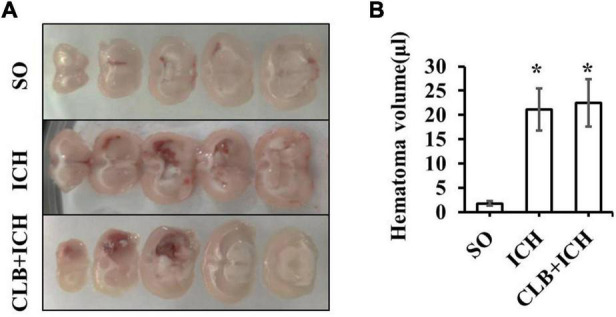
The hematoma volume of the three groups. **(A)** The brain hematoma were successfully induced 3 day after collagenase IV injection in ICH and CLB + ICH group. **(B)** There was no significant difference in hematoma volume between the ICH and CLB + ICH group 3 days after ICH induction (*p* > 0.05). *P < 0.05, compare to SO group. SO group, *n* = 5; ICH group, *n* = 5; and CLB + ICH group, *n* = 5.

**FIGURE 4:**
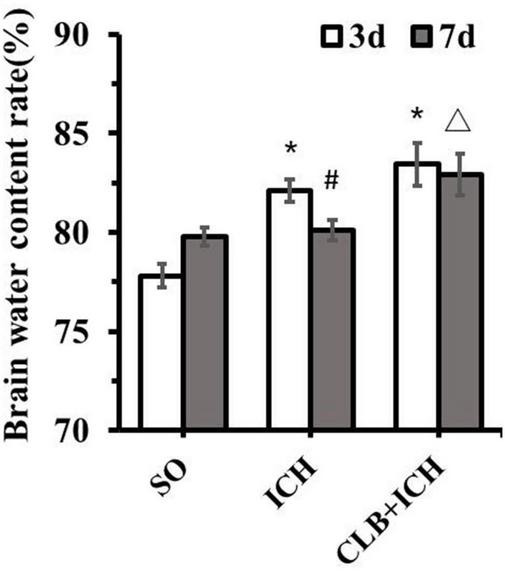
Brain water content of the three groups. There was a significant increase in the brain water content of ipsilateral cortex of the ICH group and CLB + ICH group compared to that in the sham group (*P < 0.05). Seven days after ICH, CLB significantly inhibited regression of ICH-induced cerebral edema (△, *P* < 0.05). There was a significant decrease in the brain water content for rats in the ICH group (#, *P* < 0.05), but there was no significant difference in the CLB + ICH group (*P* > 0.05). SO group, *n* = 5. ICH group, *n* = 5; and CLB + ICH group, *n* = 5, 3 or 7 days after ICH.

Since ICH mainly presents as hematoma and cerebral edema, we determined the effect of CLB on hematoma volume and cerebral water content after induction of ICH. There was no statistical difference in the hematoma volume between the ICH group and CLB + ICH group (22.4 ± 3.2 μl vs. 23.6 ± 4.7 μl, *P* > 0.05, Figures 3A,B). In addition, brain water content was assessed on 3 and 7 d after ICH. The amount of brain water content in the ipsilateral cortex of rats in the ICH group was higher compared to that in the sham group 3 d after ICH (82.1 ± 0.4% vs. 77.8 ± 0.3%, *P* < 0.05, Figure 4). However, there was no significant difference in brain water content between the CLB + ICH group and the ICH group (83.2 ± 0.5% vs. 82.1 ± 0.4%, *P* < 0.05, Figure 4). Interestingly, 7 days after ICH, brain water content in the CLB + ICH group was significantly higher than that in the ICH group (82.9 ± 0.5% vs. 80.1 ± 0.2%, *P* < 0.05, Figure 4). There was a significant decrease in the brain water content 7 d after ICH compared to 3 d after ICH (80.1 ± 0.2% vs. 82.1 ± 0.4%, *P* < 0.05, Figure 4), but there was no change in the CLB + ICH group (83.2 ± 0.5% vs. 82.9 ± 0.5%, *P* > 0.05, Figure 4). The above results indicated that CLB aggravated ICH-induced perihematomal brain edema.

### Cerebral Lymphatic Blocking Increased Inflammatory Cells in Perihematomal Tissues After Intracerebral Hemorrhage

**FIGURE 5:**
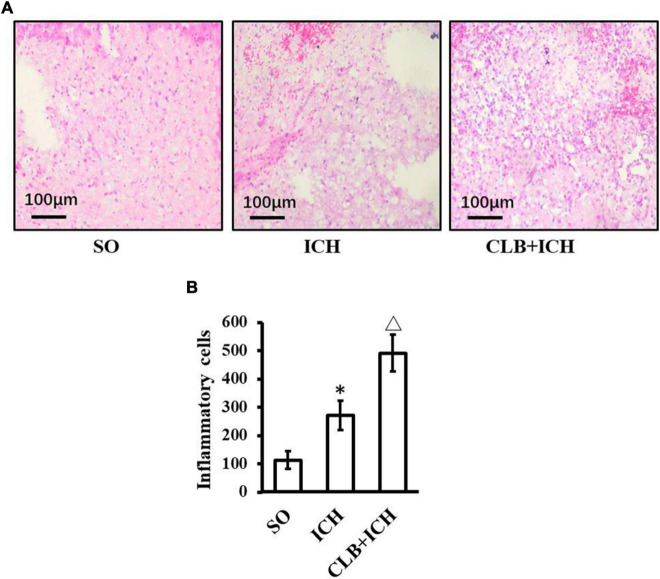
HE staining result of three groups. **(A)** Histopathological changes in perihematomal tissues of each group 3 days after ICH induction (HE staining, ×400). In the SO group, cerebral hematoma, edema, and liquefactive necrosis were not found. In the ICH group, tissues surrounding the cerebral hematoma showed edema, liquefactive necrosis, deposition of hemosiderin, inflammatory cells (mainly neutrophils). In the CLB + ICH group, there was greater edema and higher infiltration of immune cells (predominant monocytic and a low-degree neutrophilic granulocyte) in the perihematomal region compared to that in ICH group. Scale bar: 100 μm. **(B)** The number of inflammatory cells in the ICH group was significantly higher than those in control group (^∗^*P* < 0.05). Compared to ICH groups, numbers of inflammatory cells in CLB + ICH group was further increased (△, *P* < 0.05). SO group, *n* = 5; ICH group, *n* = 5; and CLB + ICH group, *n* = 5.

HE staining was used to assess any histopathological changes and accumulation of inflammatory cells in perihematomal tissues of each group 3 days after ICH induction (Figure 5). In the SO group, there was no cerebral hematoma, edema, and liquefactive necrosis observed. On the other hand, in the ICH group, the tissues surrounding the cerebral hematoma showed edema, liquefactive necrosis, deposition of hemosiderin, inflammatory cells (mainly neutrophils) infiltration and neuronal degeneration and necrosis. In the CLB + ICH group, the perihematomal region showed edema and necrosis with infiltration of cells (predominant monocytic and a low-degree neutrophilic granulocyte), which was worse compared to that in the ICH group. ICH increased the number of inflammatory cells in the perihematomal area (268.4 ± 65.2 cells/mm^2^ vs 110.7 ± 50.9 cells/mm^2^, *n* = 5, respectively). The degree of infiltration by inflammatory cells in the CLB + ICH group (488.5 ± 73.8 cells/mm^2^, *n* = 5) was significantly higher than that in the ICH group (268.4 ± 65.2 cells/mm^2^, *n* = 5). Thus, CLB aggravated histopathological damage and accumulation of inflammationary cells.

### The Effect of Cerebral Lymphatic Blocking on the Levels of Cerebral Inflammatory Cytokines in Rat With Intracerebral Hemorrhage

**FIGURE 6:**
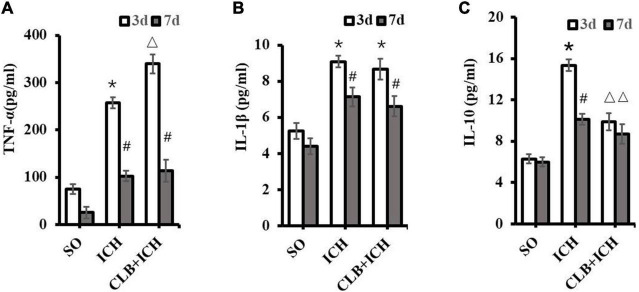
Levels of TNF-α, IL-1β, and IL-10 in the perihematoma tissue using ELISA. **(A)** Compared to SO group, TNF-α significantly increased in ICH and CLB + ICH groups (*P < 0.05) 3 days after ICH. The levels of TNF-α expression 7 days after ICH decreased significantly compared to the levels at day 3 days, in the ICH and CLB + ICH groups (#, *P* < 0.05). Compared to that in ICH group, TNF-α was significantly up-regulated in the CLB + ICH group 3 days after ICH (△, *P* < 0.05). There were no statistical difference between ICH group and CLB + ICH group 7 days after ICH. **(B)** The expression levels of IL-1β in both ICH and CLB + ICH groups were significantly higher compared to the SO group (*P < 0.05). IL-1β was significantly decreased 7 days after ICH compared to 3 days after ICH in the ICH and CLB + ICH groups (#, *P* < 0.05). There were no statistical differences in the levels of IL-1β between ICH group and CLB + ICH group 3 and 7 d after ICH (*P* > 0.05). **(C)** The expression levels of IL-10 in the CLB + ICH group were significantly lower than those in the ICH group 3 and 7 d after ICH (△, *P* < 0.05). There was a statistically significant decrease in the expression levels of IL-10 in the ICH group between 3 and 7 d after ICH (#, *P* < 0.05). However, the levels were only slightly decrease in the CLB + ICH group (*P* > 0.05). SO group, *n* = 5. ICH group, *n* = 5; and CLB + ICH group, *n* = 5, 3, or 7 days after ICH.

To determine if the glymphatic drainage system participates in the inflammatory response after ICH, the proinflammatory cytokines (TNF-α and IL-1β) and anti-inflammatory cytokine (IL-10) were assayed. The expression levels of TNF-α in brain tissues of rats in the ICH group were significantly higher than those in the SO group 3 d after ICH induction (257.8 ± 32.9 pg/ml vs. 75.0 ± 20.3 pg/ml, *P* < 0.05, Figure 6A). Similarly, the expression levels of TNF-α in the brain of rats in the CLB + ICH group were significantly higher than those in the ICH group 3 d after ICH induction (339.6 ± 30.5 pg/ml vs. 257.8 ± 32.9 pg/ml, *P* < 0.05, Figure 6A). At 7 d after ICH induction, there were no statistical difference between the ICH group and CLB + ICH group (102.3 ± 36.1 pg/ml vs. 113.6 ± 29.0 pg/ml, *P* > 0.05, Figure 6A).

At 3 d after ICH induction, there was a significant increase in the expression levels of IL-1β in both the ICH and CLB + ICH group (9.09 ± 0.22 pg/ml and 8.68 ± 0.43 vs. 5.25 ± 0.32 pg/ml, *P* < 0.05, Figure 6B). There was no statistical difference in the expression levels of IL-1β between the ICH group and CLB + ICH group 3 or 7 d after ICH induction (9.09 ± 0.22 pg/ml vs. 8.68 ± 0.43 pg/ml, 7.13 ± 0.26 pg/ml vs. 6.62 ± 0.35 pg/ml, *P* > 0.05, Figure 6B). However, the expression levels of IL-10 were significantly lower in the CLB + ICH group compared to the ICH group 3 and 7 d after ICH induction (15.34 ± 0.29 pg/ml vs. 9.85 ± 0.38 pg/ml, 10.10 ± 0.31 pg/ml vs. 8.86 ± 0.25 pg/ml, *P* < 0.05, Figure 6C). There was a significant decrease in the expression levels of IL-10 in the ICH (15.34 ± 0.29 pg/ml vs. 10.10 ± 0.31 pg/ml, *P* < 0.05) and CLB + ICH groups (9.85 ± 0.38 pg/ml, vs. 8.86 ± 0.25 pg/ml, *P* > 0.05) 7 d compared to 3 d after ICH induction (Figure 6C). These results showed that CLB increased the levels of TNF-α and reduced the levels of IL-10, which increases the ICH-induced imbalance between pro-inflammatory and anti-inflammatory cytokines in rats.

### Cerebral Lymphatic Blocking Induced Lower Aquaporins-4 and Higher Glial Fibrillary Acidic Protein in Rats With Intracerebral Hemorrhage

**FIGURE 7:**
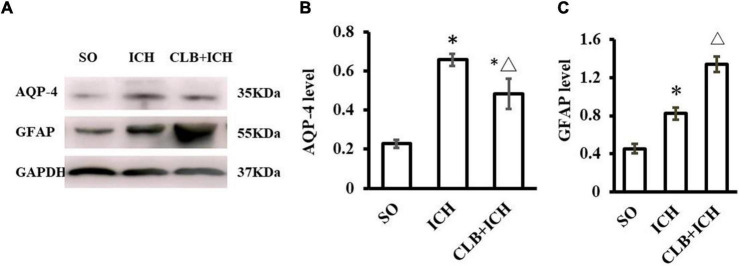
Western blot results of AQP4 and glial fibrillary acidic protein (GFAP) of three groups 3 d after ICH. **(A)** Representative result of Western Blot. **(B)** The expression of AQP4 in the ICH and CLB + ICH groups was significantly higher compared to SO group (^∗^*P* < 0.05). AQP-4 level was significantly decreased in the CLB + ICH group compared to the ICH group (△, *P* < 0.05). **(C)** The expression of GFAP was significantly increased in the ICH and CLB + ICH group as compared to the SO group (^∗^*P* < 0.05). The expression of GFAP was further increased in the CLB + ICH group compared to that in ICH group (△, *P* < 0.05). SO group, *n* = 5; ICH group, *n* = 5; and CLB + ICH group, *n* = 5.

The function of the glymphatic system relies on the AQP4 on the endfoot of astrocytes at peri-vascular areas. We wanted to determine if CLB exacerbated cerebral edema and pro-inflammatory cytokines by interfering with the functions of AQP4 and astrocytes. The Western blot results showed that the expression of AQP-4 in the ICH group was significantly increased compared to the SO group 3 d after ICH induction (*P* < 0.05, Figures 7A,B). However, the expression levels of AQP-4 were significantly lower in the CLB + ICH group compared to the ICH group (*P* < 0.05, Figures 7A,B). The expression of GFAP was significantly higher in the ICH group compared to the SO group (*P* < 0.05, Figures 7A,C). The expression of GFAP was also higher in the CLB + ICH group compared to the ICH group (*P* < 0.05, Figures 7A,C). The above results showed that CLB induced lower AQP-4 and higher GFAP levels after ICH in rats, which might influence cerebral edema and exacerbate inflammatory response.

## Discussion

The glymphatic system is a highly organized fluid transport system in which convective flow drives exchange of interstitial fluid (ISF) with cerebrospinal fluid (CSF) (Valnes et al., 2020). Studies have proved that it is a network of perivascular tunnels wrapped by astrocyte endfeet (Hablitz et al., 2020) and perivascular astrocyte water channel aquaporin-4(AQP-4) plays an important role in the exchange of CSF with ISF (Nakada and Kwee, 2019). Since it was first reported in 2012, it has been proved to be associated with central nervous system diseases, such as cerebral infarction, Alzheimer’s disease, multiple sclerosis and subarachnoid hemorrhage (Reeves et al., 2020; Chen et al., 2021). However, its function on intracerebral hemorrhage (ICH) is poorly known and our objective of this study is to investigate the role of the glymphatic function in ICH in rats.

The flow-out pathway of glymphatic system includes: (1) along the microvasculature and large draining veins to cerebral vascular; (2) along the internal cerebral and caudal rhinal veins to arachnoid granulations; and (3) along the meningeal lymph-vessel to the cervical lymph node. The ligation of the cervical lymph nodes could partially inhibit the flow and function of the glymphatic system. Our and others’ previous studies have confirmed that blocking glymphatic drainage can aggravate the injury and apoptosis of neurons after SAH in rats (Sun et al., 2009; Wang et al., 2010; Xi Chang et al., 2010), indicating “lymphatics of the brain” may play an endogenous protective role in SAH. To ICH, the results of this study clearly revealed that blocking the glymphatic drainage exacerbated the neuronal apoptosis of peri-hemorrhage in rats by up-regulating Caspase-3 expression and neurological impairment after ICH. Therefore, the glymphatic system provides protective function in ICH.

It was reported that the clot clearance after subarachnoid hemorrhage was blocked when the glymphatic system was ablated (Chen J. et al., 2020). However, in this experiment, it was observed that cerebral glymphatic blocking only aggravate the edema around the hematoma, but not increase the volume of the hematoma. It may be due to the special anatomical structure such as smaller perivascular space in the parenchyma of the brain than subarachnoid area, which cannot directly drain intracranial hematoma out of the brain. The glymphatic system may also accelerate hematoma absorption, but the absorption process is slow and not easily observed in a short period of time (Liu et al., 2016).

Based on the structural basis of the glymphatic system (Huang et al., 2021), the above protective function of the glymphatic drainage should not be realized through direct action on neurons, but through other probable pathways. “Lymphatics of the brain” drains fluid and solutes from the brain by perivascular pathway (Carare et al., 2008). The Glymphatic system of the brain may contribute to partial immune privilege of the brain and play a role in neuro-immunological diseases (Weller et al., 2009). To evaluate the changes in expression of inflammatory cytokines, the numbers of inflammatory cells and several markers including TNF-α, IL-1β, and IL-10 were detected in this study.

TNF-α was elevated in ICH patients and increased peri-hematoma. TNF-α expression contributed to edema development after ICH in rodents (Rendevski et al., 2018). The expression levels of TNF-α peaked 3 d after ICH, and this increase aggravated secondary neurological impairment after ICH (Zhang et al., 2019). In this study, the expression levels of TNF-α were determined 3 and 7 d after ICH induction. There was an increase in the expression of TNF-α in the peri-hematoma after ICH induction, which was increased further by CLB. The difference between the CLB + ICH group and ICH group gradually decreased 7 days after ICH. This result was also consistent with the changes in ICH-induced cerebral edema. IL-1β is a known pro-inflammatory cytokine that induces cerebral inflammatory reaction and is associated with the development of cerebral edema in ICH rats. It has been shown that IL-1β is released from activated microglia 1 h after ICH induction and reaches peak concentration 3 d after ICH (Wei et al., 2014). However, there was no increase in the expression of IL-1β in rats with CLB, which suggests that IL-1β was not associated with the CLB-induced aggravation of neurological impairment resulting from ICH. Previous studies have shown that IL-10 protects the nerve cells of peri-hematoma after ICH (Ewen et al., 2013; Zhou et al., 2017), and that the up regulation of IL-10 alleviates neurological deficits in patients with ICH (Ewen et al., 2013). In this study, CLB inhibited the expression of IL-10 3 d after ICH. GFAP is a marker of reactive astrocytes and it was significantly increased after CLB under the change of TNF-α and IL-10. Therefore, the inhibition of the glymphatic drainage aggravated the imbalance between pro-inflammatory and anti-inflammatory cytokines and induced astrocyte activation after ICH.

Perivascular astrocyte endfeet is the main constituent of the glymphatic drainage and AQP-4 on it facilitates the transport of interstitial fluid in the brain to eliminate toxic factors (Liu et al., 2020), making AQP-4 as the most important element in glymphatic system. Previous studies reported that AQP-4 deletion in mice increased edema and altered the integrity of the blood-brain barrier following ICH, as well as aggravated early brain injury following SAH through impairment of the glymphatic system (Chu et al., 2014; Liu et al., 2020). Similar to findings of a previous study (Tang et al., 2010), there was an increase in the expression of AQP-4 after ICH in rats. Interestingly, the significant increase in AQP-4 expression following ICH was inhibited by CLB, which also explained why CLB aggravated cerebral edema and the accumulation of toxic factors after ICH in rats.

It was notably that AQP4-knockout inhibited edema and improved neurological outcome in the ischemic stroke or acute water intoxication model, which seems to be contradictory to results in SAH (Liu et al., 2020) or ICH described here. However, it is understandable that AQP4 mediates the bidirectional water flux, facilitating water influx in the evolution of cytotoxic edema during early ischemia and water efflux in vasogenic edema during late ischemia or cerebral hemorrhage (Vella et al., 2015). Expression of AQP4 increased after ICH and the increment was inhibited after CLB, which suggested only CLB for 3 days could inhibit the AQP4 expression. However, no difference of AQP4 expression was reported after CLB for 2 week (Cao et al., 2018) and it increased after CLB for 4 weeks (Wang et al., 2019) or 6 weeks (Zou et al., 2019). The time-course impact of CLB on AQP4 expression needs further investigation. Meanwhile, the inflammatory balance and AQP4 function regulated by glympahtic system are interactive, as detrimental inflammation causes reactive astrocyte, release matrix metallo-proteinases (MMPs) and the subsequent disturbed AQP4 expression or location (Candelario-Jalil et al., 2009), and as AQP4 participants in inflammation response *via* edema resolution (Fukuda and Badaut, 2012).

Notably, unlike AQP4, the GFAP significantly increased after CLB compared to ICH group. GFAP is astrocytic cytoskeletal element and a major contributor of astrocytic reactivity, and abnormal expression of GFAP occurs in neuroinflammation and brain edema-eliciting diseases (Li et al., 2020). The imbalance between pro-inflammatory and anti-inflammatory cytokines and increased edema after CLB leads to increased GFAP expression. AQP4 expression is reported to be in parallel with the level of GFAP (Wang and Hatton, 2009). After cerebral ischemia, the expressions of AQP-4 and GFAP are upregulated, and the foot processes of astrocytes swell (Filchenko et al., 2020). The mechanism of CLB inhibiting AQP4 expression after ICH needs to be further investigated. However, the depletion of AQP4 does not consistently influence the expression level of GFAP and the distribution of GFAP change dramatically following changes in the water flux/volume in astrocytes (Nicchia et al., 2016).

In addition, the only CLB group was not designed in this study. However, only CLB was reported to reduce spontaneous motor activity in the open field test and motor coordination ability in rotarod testing; increase the IL-1β, TNF-α, and GFAP expression; and influence the AQP4 expression (Cao et al., 2018; Wang et al., 2019; Zou et al., 2019). The neuronal apoptosis was not obvious after only CLB (Cao et al., 2018; Wang et al., 2019) and it might be related to the short time interval after CLB. Therefore, the exacerbation of neurological impairment, cerebral edema and neuroinflammation in CLB + ICH group is due to additive and/or synergistic effect.

The findings of our study indicated that CLB increased cerebral edema in rats after ICH by up-regulating TNF-α and down-regulating AQP-4. In addition, CLB increased inflammatory damage after ICH in rats by increasing TNF-α and inhibiting IL-10 expression. Therefore, CLB exacerbated neuronal apoptosis and neurological deficits in rats with ICH. Our study findings also demonstrated that the glymphatic drainage provides neuroprotection against brain edema, neuroinflammation and neuronal apoptosis after ICH under normal physiological conditions. The regulation of the glymphatic function might be beneficial for ICH treatment and prognosis. There are some limitations in our research. The status of the glymphatic system after ICH and its neurological protective function in ICH was not directly investigated. The signaling pathways involved in CLB-induced imbalance between proinflammatory and anti-inflammatory cytokines have not been studied. Therefore, further investigation is required.

## Data Availability Statement

The original contributions presented in the study are included in the article/supplementary material, further inquiries can be directed to the corresponding author.

## Ethics Statement

The animal study was reviewed and approved by the Committee for Animal Experiments at Taizhou Hospital of Zhejiang Province in China.

## Author Contributions

XL, GW, and SK contributed to the conception and design of the study. XL, GW, NT, LL, CL, and FW implemented the research. XL and GW wrote the first draft of the manuscript. SK reviewed and edited the manuscript. All authors contributed to manuscript revision, read, and approved the submitted version.

## Conflict of Interest

The authors declare that the research was conducted in the absence of any commercial or financial relationships that could be construed as a potential conflict of interest.

## Publisher’s Note

All claims expressed in this article are solely those of the authors and do not necessarily represent those of their affiliated organizations, or those of the publisher, the editors and the reviewers. Any product that may be evaluated in this article, or claim that may be made by its manufacturer, is not guaranteed or endorsed by the publisher.
